# Suppression of Bcl3 Disrupts Viability of Breast Cancer Cells through Both p53-Dependent and p53-Independent Mechanisms via Loss of NF-κB Signalling

**DOI:** 10.3390/biomedicines12010143

**Published:** 2024-01-10

**Authors:** Daniel J. Turnham, Hannah Smith, Richard W. E. Clarkson

**Affiliations:** European Cancer Stem Cell Research Institute, School of Bioscience, Cardiff University, Cardiff CF24 4HQ, UK

**Keywords:** Bcl3, senescence, NF-κB, breast cancer, p53, SASP, apoptosis

## Abstract

The NF-κB co-factor Bcl3 is a proto-oncogene that promotes breast cancer proliferation, metastasis and therapeutic resistance, yet its role in breast cancer cell survival is unclear. Here, we sought to determine the effect of Bcl3 suppression alone on breast cancer cell viability, with a view to informing future studies that aim to target Bcl3 therapeutically. Bcl3 was suppressed by siRNA in breast cancer cell lines before changes in viability, proliferation, apoptosis and senescence were examined. Bcl3 suppression significantly reduced viability and was shown to induce apoptosis in all cell lines tested, while an additional p53-dependent senescence and senescence-associated secretory phenotype was also observed in those cells with functional p53. The role of the Bcl3/NF-κB axis in this senescence response was confirmed via siRNA of the non-canonical NF-κB subunit NFKB2/p52, which resulted in increased cellular senescence and the canonical subunit NFKB1/p50, which induced the senescence-associated secretory phenotype. An analysis of clinical data showed a correlation between reduced relapse-free survival in patients that expressed high levels of Bcl3 and carried a p53 mutation. Together, these data demonstrate a dual role for Bcl3/NF-κB in the maintenance of breast cancer cell viability and suggests that targeting Bcl3 may be more beneficial to patients with tumours that lack functional p53.

## 1. Introduction

Apoptosis and senescence are two well-documented processes with distinct outcomes that help maintain controlled cell turnover; however, in cancer these fail-safe mechanisms are often compromised, resulting in uncontrolled tumour growth [1,2]. While the controlled elimination of supernumerary cells through apoptosis is well understood, the effectiveness of senescence, which drives the affected cell into a state of dormancy, is less well characterised and controversial. Evidence of reversibility and a pro-tumourigenic impact on the local microenvironment has led to some debate as to whether or not the induction of senescence in tumour cells is a desirable outcome for cancer therapy [3,4,5]. Many therapies designed to target tumour cells trigger either apoptosis or senescence as an indirect response to the cellular stress placed on the cells [1,6]. One well-studied example of this is the use of chemotherapeutic agents, which although are primarily designed to target cell replication, have been demonstrated to induce both apoptosis and senescence in tumours (reviewed in [7,8]). Consistent with most other solid tumours, in breast cancer, p53 is frequently mutated and is thought to play a critical role in determining this response to chemotherapy. In tumours maintaining functional wild-type p53 (p53^wt^), chemotherapy often drives a senescence phenotype that has been shown to limit the clinical outcome when compared to the typical apoptotic response observed in mutant p53 (p53^mut^) tumours [9,10,11]. Improved understanding of the molecular drivers of these differential responses as well as uncovering novel, safe ways to stably disrupt tumour cell survival, and thus eliminating the potentially adverse effects of long-term tumour dormancy could provide patients with sustained therapeutic responses. 

The NF-κB family of transcription factors are an attractive clinical target due to the known role that this family plays in maintaining tumour survival through regulating both apoptosis and senescence in breast cancer [12,13,14,15]. Targeting the NF-κB pathway has been of clinical interest for many years given its established role in driving tumour progression; however, so far, attempts to generate targeted NF-κB inhibitors for clinical use have failed, largely due to toxicity issues as a result of the multifaceted roles of the numerous NF-κB subunits in normal physiology [16]. The directed modulation of specific NF-κB pathways may represent a more tangible approach rather than global NF-κB inhibition. One such approach of NF-κB modulation has been suggested through targeting the B-cell lymphoma 3-encoded protein (Bcl3), which is an atypical IkB family member that regulates both canonical and non-canonical NF-κB signalling through its interactions with p50 and p52 homodimers, respectively [17,18]. Bcl3 has been implicated with a role in tumour progression and is activated alongside NF-κB subunits p50 and p52 in breast cancer [19]; however, Bcl3 knockout mice remain viable with minimal adverse effects and show increased apoptosis in mammary glands, making it an attractive therapeutic target [20,21]. 

Despite Bcl3’s well-recognised association with metastatic disease, recently attributed to its ubiquitous role in cancer cell migration, there is little evidence directly linking Bcl3 with the NF-κB mediated regulation of basal survival mechanisms in breast cancer [22,23,24]. Bcl3 is known to regulate the NF-κB mediated transcription of genes associated with both apoptosis and proliferation such as Bcl-2, Cyclin D1 and STAT3 [25,26,27,28], and has been implicated with maintaining breast cancer cell survival following DNA damage through both p53-dependent and -independent pathways [29,30]. The importance of Bcl3 in maintaining tumour cell survival in the absence of exogenous stress inducers, however, remains unclear, with transgenic ErbB2 and triple-negative mouse models showing little effect on primary tumour growth following Bcl3 inhibition [22,23]. On the other hand, Bcl3 overexpression has been shown to promote tumour establishment and growth in ER-positive MCF-7 xenografts [31], while recently a small-molecule inhibitor of Bcl3 has shown efficacy in reducing tumour growth in triple-negative xenografts [32]. It has been suggested that other cancer types may become ‘addicted’ to Bcl3 and that a loss of Bcl3 can specifically disrupt tumour cell survival, further adding to the appeal of targeting Bcl3 therapeutically [33,34]. 

Here, we set out to determine the role of Bcl3 in the maintenance of breast cancer cell survival in the absence of chemotherapeutic challenge. The suppression of Bcl3 expression significantly reduced viability across a panel of ER-positive breast cancer cell lines, primarily through a significant increase in apoptosis. An additional senescence phenotype was observed in a subset of p53 wildtype cells, which was demonstrated to be dependent on p53 and caused via a disruption to downstream NF-κB signalling. Interestingly, high Bcl3 expression was shown to correlate with reduced relapse-free survival (RFS) in breast cancer patients with tumours harbouring p53 mutations, suggesting that these patients may benefit most from Bcl3 targeting therapies. Together, these data show for the first time that Bcl3 plays a critical role in the (steady-state or homeostatic) survival of breast cancer cells, mediated through p53-dependent and p53-independent mechanisms. This has important implications for predicting therapeutic responses to targeted Bcl3 inhibition in breast cancer.

## 2. Materials and Methods

### 2.1. Cell Lines and Reagents

The human breast cancer BT474 cell line was obtained directly from ATCC, while MCF-7, ZR751 and T47D cells were previously obtained from ATCC and provided as a kind gift from Dr. Julia Gee (Department of Pharmacy, Cardiff University, Cardiff, UK). Each cell line was maintained in RPMI 1640 GlutaMAX media (Invitrogen) supplemented with 10% foetal bovine serum (Invitrogen) at 37 °C in 5% CO_2_. 

ON-Target plus SMART pool siRNAs (Dharmacon, Cambridge, UK), containing a pool of 4 different individual siRNA constructs each designed to inhibit their respective targets, Bcl3 (#L-003874-00-0005), NFkB1 (#L-003520-00-0005), NFkB2 (#L-003918-00-0005) and p53 (#L-003329-00-0005), were used, while a pool of 4 non-targeting control siRNAs was used as a control (#D-001810-10). Transfection was performed on adherent cells using lipofectamine RNAiMax (Invitrogen) and serum-free Opti-MEM (Invitrogen) in accordance with the manufacturer’s instructions. For experiments that used two siRNA pools together, additional scRNA was added to single transfection control mixes to account for additional RNA.

### 2.2. Cell Viability Assays

In order determine end-point cell viability, cell titre blue (Promega, Southampton, UK) was added to each well following manufacturer’s instructions and incubated for 1.5 h, before the fluorescence of each well was measured using a Clariostar plate reader. All stats were performed on the raw data but were presented by normalising to scRNA controls to give relative viability.

### 2.3. Proliferation and Apoptosis Assays

Proliferation and apoptosis assays were performed using an IncuCyte real-time analysis system (Essen Bioscience, Hertfordshire, UK). Cells were grown in 96-well plates and supplemented with Annexin V or caspase 3/7 green reagent (Essen Bioscience) at the time of siRNA transfection, which emit a green, fluorescent signal upon binding to apoptotic cells. Cells were maintained within the IncuCyte system for the duration of experiments and analysed using the IncuCyte live-cell-imaging software. Cell confluency was determined using IncuCyte confluence analysis of phase-contrast images, while additional masks were set to detect positive-fluorescent signals, with each mask adapted for each cell line to compensate for differences in morphology. 

### 2.4. Senescence-Associated β-Gal Assay

The senescence β-galactosidase (SA-β-gal) staining kit (Cell Signalling) was used to determine cellular senescence following treatments. Cells grown in a 12-well plate format were prepared and fixed for 15 min in accordance with the manufacturer’s instructions before being incubated overnight at 37 °C in freshly prepared SA-β-gal detection solution. SA-β-gal activity was assessed by counting the number of positively stained cells using bright-field microscopy (Leica).

### 2.5. Immunofluorescence

To determine the number of actively dividing cells, immunofluorescence staining against ki67 (SolA15-Merck) and pH3 (Ser10- Cell Signalling) was performed. Cells grown on glass coverslips were fixed, permeabilised and blocked in 5% BSA before being stained with primary antibody overnight at 4 °C. Fluorescently labelled secondary antibodies raised against rat or rabbit (Abcam, Cambridge, UK) were added to cells for 1 h at room temperature, with DAPI. Cover slips were then mounted onto glass slides and imaged.

### 2.6. qRT-PCR Analysis

Gene expression analysis was performed using RNA extracted from an RNeasy mini kit (Qiagen) following the manufacturer’s instructions before cDNA was generated using the QuantiTect reverse transcription kit (Qiagen, Manchester, UK). Multiplex qRT-PCR reactions were performed using a QuantStudio 7 Real-Time PCR machine (Applied Biosystems, Altrincham, UK) using predesigned TaqMan primers (ThermoFisher Scientific, Altrincham, UK) designed to target Bcl3 (Hs00180403_m1), CDKN1A (p21) (Hs00355782_m1), CDKN2B (p15) (Hs00793225_m1), IL-6 (Hs00985639_m1), IL-8 (Hs00174103_m1), CXCL10 (Hs01124251_g1), BBC3 (PUMA) (Hs00248075_m1) and ACTB (Hs99999903_m1). TaqMan Universal Master Mix II (ThermoFisher Scientific) and template cDNA were used with the following thermocycling conditions: 95 °C for 10 min, followed by 40 cycles of 95 °C for 15 s, and 60 °C for 1 min. 

### 2.7. KM-Plotter Data Analysis

All patient data analysis was performed using KM plotter gene chip breast cancer cohorts [35], using the mean expression of Bcl3 specific probes 204907_s_at and 204908_s_at, with patients split into Bcl3-high and -low expression using the median expression value for each patient cohort. For quality control, redundant samples were removed and biased arrays were excluded as previously described [36,37]. RFS was determined for patient groups with no previous systemic treatments and for patients that had received any form of endocrine therapy or chemotherapy.

### 2.8. Statistics

Error bars on all graphs represent the standard error of the mean from least 3 independent biological repeats with statistical analysis performed in Graphpad prism (Graphpad prism 10 software, Boston, MA, USA) using an unpaired student’s *t*-test for two-way comparisons or a one-way ANOVA for comparing multiple datasets as stated in each figure legend. RFS analysis was performed on KM-plotter, which automatically calculates statistical significance using log-rank test as previously described [35]. 

## 3. Results

### 3.1. Suppression of Bcl3 Causes a Significant Loss of Viability in Breast Cancer Cell Lines 

The anti-metastatic effects of Bcl3 suppression have been well documented; however, the consequences of suppressing Bcl3 on breast cancer viability, other than in response to DNA damage, remain poorly defined. High levels of Bcl3 are known to reduce RFS in patients with ER-positive breast cancer [38]; therefore, to elucidate the role of Bcl3 in regulating breast cancer cell survival, we began by assessing the effect of Bcl3 suppression in a range of ER-positive breast cancer cell lines expressing either wildtype or mutant forms of p53. 

**Figure 1:**
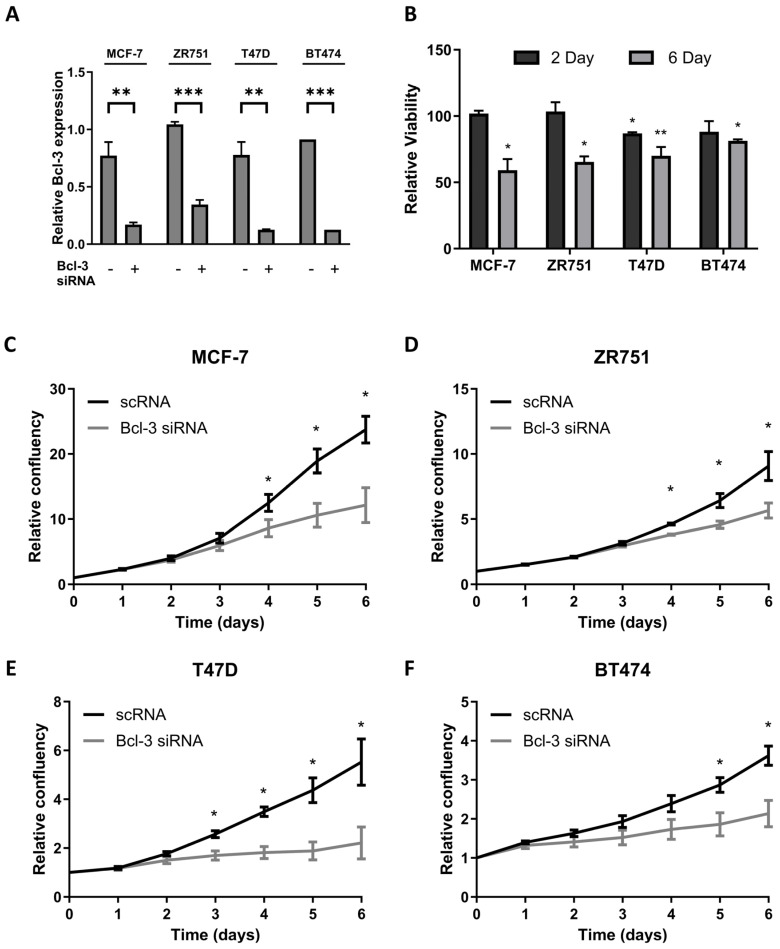
Bcl3 suppression reduces cell confluency over time. (**A**) Relative Bcl3 expression 6 days post-Bcl3 siRNA transfection in 4 breast cancer cell lines was assessed using qRT-PCR. (**B**) Cell titre blue was used to assess cell viability following 2 and 6 days of siRNA treatment, displayed as relative viability loss compared to that of scRNA controls. (**C**–**F**) IncuCyte analysis software (version 2022B) was used to analyse differences in cell confluency in (**C**) MCF-7, (**D**) ZR751, (**E**) T47D and (**F**) BT474 cells following Bcl3 suppression with data normalised to the confluency at the time of siRNA transfection. Error bars represent ± SEM of *n* = 3 minimum, with statistical differences determined using a 2-tailed *t* test; * *p* < 0.05. ** *p* < 0.01, and *** *p* < 0.001.

To determine the effects of Bcl3 inhibition on breast cancer cell survival, a pool of Bcl3-specific siRNAs were transfected into four breast cancer cell lines (p53^wt^ = MCF-7 and ZR751; p53^mut^ = T47D and BT474), before cell viability was analysed using cell titre blue in comparison to that of cells transfected with non-targeting control siRNA (scRNA). The efficient knockdown of Bcl3 expression was confirmed via qRT-PCR, with Bcl3 significantly suppressed up to 6 days post-siRNA transfection (Figure 1A). Cell viability was measured following 2 and 6 days of Bcl3 suppression with only T47D cells showing a significant reduction compared to scRNA controls at the earlier time point (Figure 1B). Following the prolonged 6-day inhibition of Bcl3, a significant reduction in viability was observed in each line (Figure 1B). These results were confirmed using IncuCyte time-lapse microscopy, which showed a significant reduction in cell confluency over a 6-day period, with these differences becoming initially apparent around 4 days of Bcl3 inhibition in each cancer cell line (Figure 1C–F). 

### 3.2. Bcl3 Loss Induces p53-Independent Apoptosis and p53-Dependent Senescence in Breast Cancer Cells

Bcl3 is known to regulate the expression of key cell death and proliferation regulators; therefore, we next sought to determine which of these processes were being disrupted following Bcl3 suppression. 

**Figure 2:**
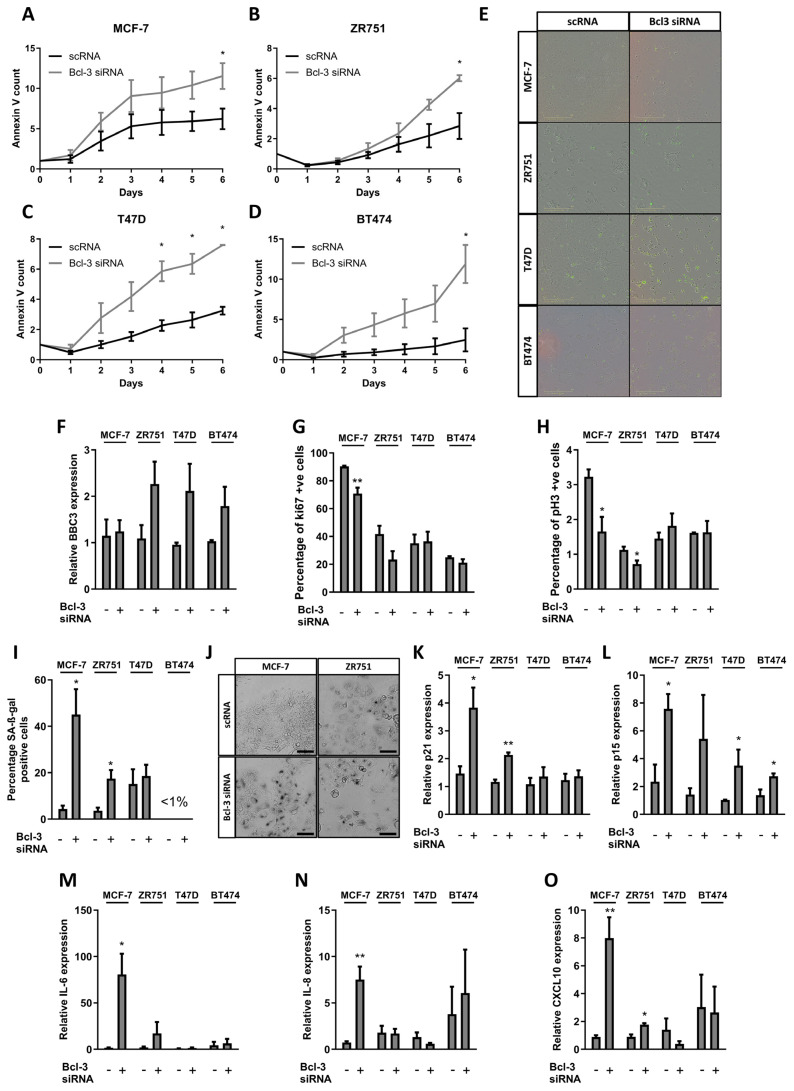
Bcl3 loss induces p53-independent apoptosis and p53-dependent senescence in breast cancer cells. Bcl3 was suppressed in breast cancer cell lines with the addition of Annexin V reagent to determine apoptosis using IncuCyte analysis software over 6 days. (**A**) MCF-7, (**B**) ZR751, (**C**) T47D and (**D**) BT474 cells each showing a significant increase in Annexin V staining; (**E**) representative images of each cell line at the endpoint. Following 6 days of siRNA, cells were harvested or fixed for further analysis. (**F**) PUMA expression determined by qRT-PCR showing an increase in three of the cell lines following Bcl3 suppression. Immunofluorescence staining for cell cycle markers (**G**) ki67 and (**H**) pH3 showed a reduction in the percentage of positively stained MCF-7 and ZR751 cells. (**I**) Senescence-associated β-gal staining was increased in Bcl3 suppressed MCF-7 and ZR751 cells, (**J**) representative images at endpoint, scale bar = 100 µm. qRT-PCR analysis showed a significant increase in (**K**) p21 expression in MCF-7 and ZR751 cells, while (**L**) p15 expression was upregulated in each cell line following Bcl3 suppression. SASP genes (**M**) IL-6, (**N**) IL-8 and (**O**) CXCL10, were also upregulated in p53 wildtype MCF-7 and ZR751 cells but not in p53-mutant T47D or BT474 cells. Error bars represent ± SEM of *n* = 3 minimum, with statistical differences determined using a 2-tailed unpaired *t* test, * *p* < 0.05. ** *p* < 0.01. Expanded versions of (**E**,**J**) are available in Supplementary Figures S1 and S3, respectively.

Real-time Annexin V staining identified a significant increase in apoptosis over 6 days, consistent with the kinetics of cell proliferation previously identified; however, the amplitude of the apoptotic response was lower in the p53^wt^ cell compared to that in its p53^mut^ counterpart lines (a fold change of Bcl-3 siRNA compared to scRNA at a 3-day timepoint: MCF-7 = 1.71, ZR751 = 1.46, T47D = 2.72 and BT474 = 4.71) (Figure 2A–E and Supplementary Figure S1). Bcl3 is known to regulate several genes that regulate apoptosis and the increase observed here following Bcl3 suppression was correlated to an upregulation in BBC3 expression in three cell lines; however, this was not significant (Figure 2F).

To determine any changes in mitosis following Bcl3 loss, changes in the expression of cell cycle markers ki67 and pH3 were assessed using immunofluorescence. No changes were observed in either p53^mut^ cell line; however, a significant reduction in both markers was observed in MCF-7 cells, with a similar trend observed in ZR751 cells, although only pH3 was significantly reduced with ki67 reduced by 44% but with larger variability observed between repeats, likely due to slight differences in transfection efficiency (Figure 2G,H and Supplementary Figure S2). Furthermore, populations of cells in the p53^wt^ cell lines exhibited morphological signs of senescence after Bcl3 suppression that were not evident in any of the p53^mut^ cell lines tested, the specificity of which was confirmed with the senescence-associated marker beta-galactosidase (SA-β-gal) (Figure 2I,J and Supplementary Figure S3). A significant increase in the proportion of SA-β-gal positive cells was observed in both MCF-7 and ZR751 cells following Bcl3 suppression while neither T47D nor BT474 p53^mut^ cell lines exhibited any increase in this marker (Figure 2I).

Cell cycle inhibition observed in senescence is typically controlled by cyclin-dependent kinase (CDK) inhibitors encoded by CDKN2A (p16), CDKN2B (p15), and CDKN1A (p21) [39]. As p16 is frequently deleted in breast cancer cell lines and not expressed in MCF-7, ZR751 or T47D cells, we sought to determine whether or not a loss of Bcl3 resulted in alterations to p15 and p21 using qRT-PCR [40,41]. Following Bcl3 suppression, p21 was significantly upregulated in both MCF-7 and ZR751 cells; however, no change was observed in p53^mut^ cell lines, suggesting this was important for the induction of senescence in p53^wt^ cells alone (Figure 2K). On the other hand, p15, which is an alternative regulator of cell cycle arrest, was upregulated in all cell lines (significantly in three of the lines including T47D and BT474), suggesting this effect was not specific to p53-mediated senescence (Figure 2L). 

Another hallmark of senescence is the appearance of the senescence-associated secretory phenotype (SASP), which involves the cell-autonomous secretion of cytokines to promote the regulation and maintenance of senescence within the tissue microenvironment. A strong SASP phenotype was observed in MCF-7 cells with IL-6, IL-8 and CXCL10 all significantly upregulated following Bcl3 suppression (Figure 2M–O). Both IL-6 and CXCL10 were also upregulated in ZR751 cells; however, only CXCL10 was significant, while no change was observed in IL-8 expression. No significant changes were observed in either p53^mut^ cell line, further suggesting the importance of p53 in both initiating and maintain senescence following a loss of Bcl3. 

Together, these observations indicated that the suppression of Bcl3 led to a universal reduction in breast cancer cell viability but that the mechanism by which this was mediated was associated with p53 status.

### 3.3. Expression of p53 Is Essential for Driving Senescence Following Bcl3 Loss

The senescence phenotype following Bcl3 suppression was only observed in breast cancer cell lines expressing wildtype p53, while a loss of viability in p53^mut^ cells was associated with the induction of apoptosis alone. Senescence observed in p53^wt^ breast cancer following chemotherapy is known to reduce clinical response while a pro-tumourigenic microenvironment driven through SASP has been observed in certain contexts of therapy-induced senescence (TIS) [9,10,11]. Therefore, elucidating the mechanisms of senescence following Bcl3 loss could have important clinical implications for targeting Bcl3. To confirm whether or not senescence was p53-dependent, Bcl3 and p53 were simultaneously suppressed in MCF-7 and ZR751 cells to determine whether or not the loss of functional p53 could rescue the senescence phenotype previously observed following Bcl3 inhibition.

**Figure 3:**
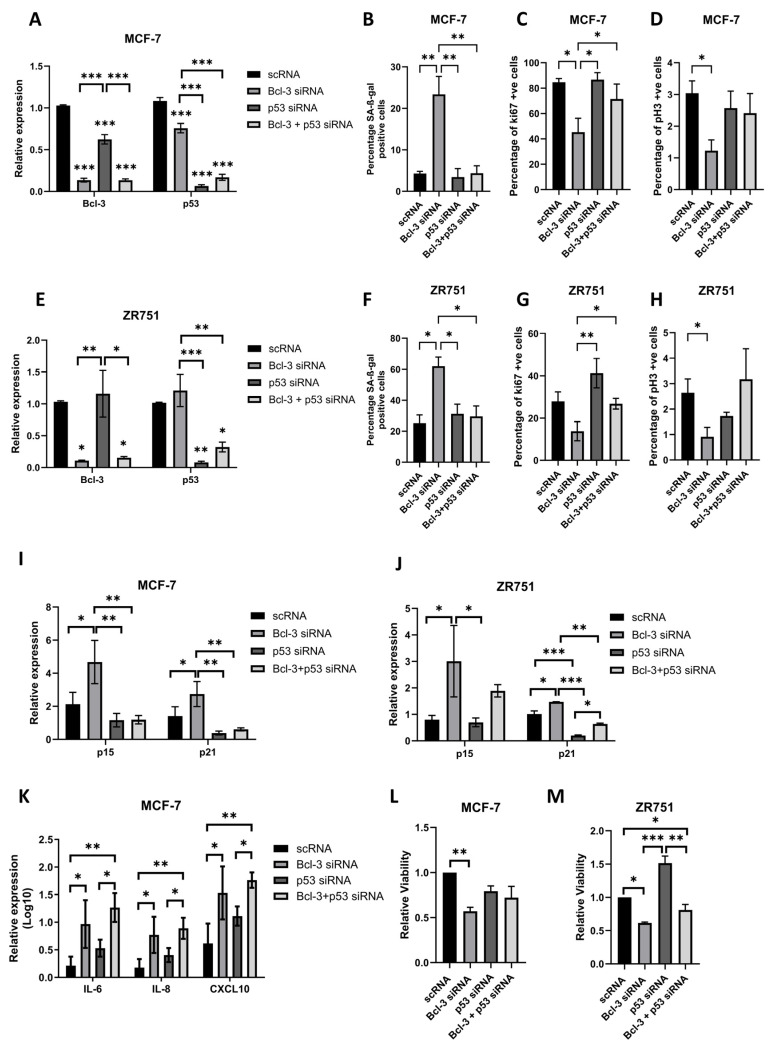
Loss of p53 rescues senescence in Bcl3-suppressed cells. Bcl3 and p53 were suppressed either alone or together in MCF-7 and ZR751 cells for 6 days. The suppression of Bcl3 and p53 was confirmed via qRT-PCR in (**A**) MCF-7 and (**E**) ZR751 cells. (**B**,**F**) Senescence-associated β-gal staining was increased following Bcl3 suppression, with no change observed when p53 was suppressed alone or with Bcl3. The percentage of cells positively stained for the cell cycle markers (**C**,**G**) ki67 and (**D**,**H**) pH3 was reduced in MCF-7 and ZR751 cells following Bcl3 inhibition, with no change following p53 inhibition alone or with Bcl3. (**I**,**J**) qRT-PCR analysis of p15 and p21 expression showed that the significant increase in p21 expression following Bcl3 inhibition was mitigated when p53 was also suppressed in both cell lines. (**K**) The expression of SASP genes IL-6, IL8 and CXCL10 was not rescued in MCF-7 cells following the combined suppression of p53 and Bcl3. Cell titre blue analysis demonstrated no further loss in cell viability in (**L**) MCF-7 or (**M**) ZR751 cells following combined Bcl3 and p53 suppression Error bars represent ± SEM of *n*= 3 with statistical differences determined using a one-way ANOVA; * *p* < 0.05, ** *p* < 0.01, and *** *p* < 0.001.

The combined knockdown of Bcl3 and p53 was confirmed in both cell lines after 6 days of siRNA-mediated suppression via qRT-PCR (Figure 3A,E). Bcl3-specific silencing in MCF-7s resulted in a significant reduction in p53 expression while p53 specific silencing resulted in a significant reduction in Bcl3 expression compared to that of scRNA; however, this was not observed in ZR751 cells. To confirm the rescue of senescence, SA-β-gal staining was performed in each cell line, with p53 suppression rescuing the previously observed increases in SA-β-gal (Figure 3B,F). This was accompanied by a rescue in the number of cells expressing proliferation markers pH3 and ki67, which was also rescued following a combination of p53 and Bcl3 suppression suggesting that p53 was mediating the previously observed cell cycle arrest (Figure 3C,D,G,H). Interestingly, the loss of p53 alone had no significant effect on the percentage of either proliferation marker, in line with previously published work [42]. A similar trend was observed following a qRT-PCR analysis of p21 expression, with simultaneous Bcl3 and p53 suppression inhibiting the increased expression of p21 when Bcl3 alone was supressed (Figure 3I,J). The expression of p15 followed a similar trend in MCF-7 cells, with a loss of p53 inhibiting the upregulation of p15 following Bcl3 suppression; however, no significant change was observed between Bcl-3 with and without p53 suppression in ZR751 cells (Figure 3I,J). This was likely due to the large variability observed between repeats and was subsequently confirmed in later figures. Interestingly, the loss of p53 was unable to inhibit the increased expression of SASP markers, with IL-6, IL-8 and CXCL10 all still significantly upregulated following combined p53 and Bcl3 siRNA treatment (Figure 3K). Together, these data suggest that functional p53 is required for the induction of senescence/cell quiescence following a prolonged loss of Bcl3 but that it is not responsible for the upregulation of accompanying SASP cytokines. 

We next sought to determine whether or not rescuing senescence through p53 suppression was sufficient to induce a more potent apoptosis affect. Cell viability was assessed 6 days post-siRNA transfection with combined Bcl3 and p53 siRNA resulting in a small non-significant increase in viability compared to that observed with Bcl3 siRNA alone, suggesting that although the population of senescent cells had been rescued, this did not increase the apoptotic response (Figure 3L,M). This response was confirmed via IncuCyte confluency, Annexin V and caspase 3/7 analysis, which showed that the addition of p53 suppression had little effect on changes in overall viability or apoptosis following Bcl3 loss (Supplementary Figure S4). Together, these data highlight the importance of p53 in initiating senescence following Bcl3 loss but suggest that it is not essential for mediating the apoptotic response.

### 3.4. Suppression of p50 and p52 Demonstrates Distinct Roles for Canonical and Non-Canonical NF-κB Signalling in Mediating Senescence

A primary function of Bcl-3 is to regulate canonical and non-canonical NF-κB signalling pathways through interactions with both p50 and p52 homodimers, respectively. Both pathways have been linked previously with regulating senescence [43,44]; therefore, we sought to determine whether or not the suppression of p50 or p52 via siRNA silencing was able to recapitulate the senescence phenotype observed following Bcl-3 inhibition. 

**Figure 4:**
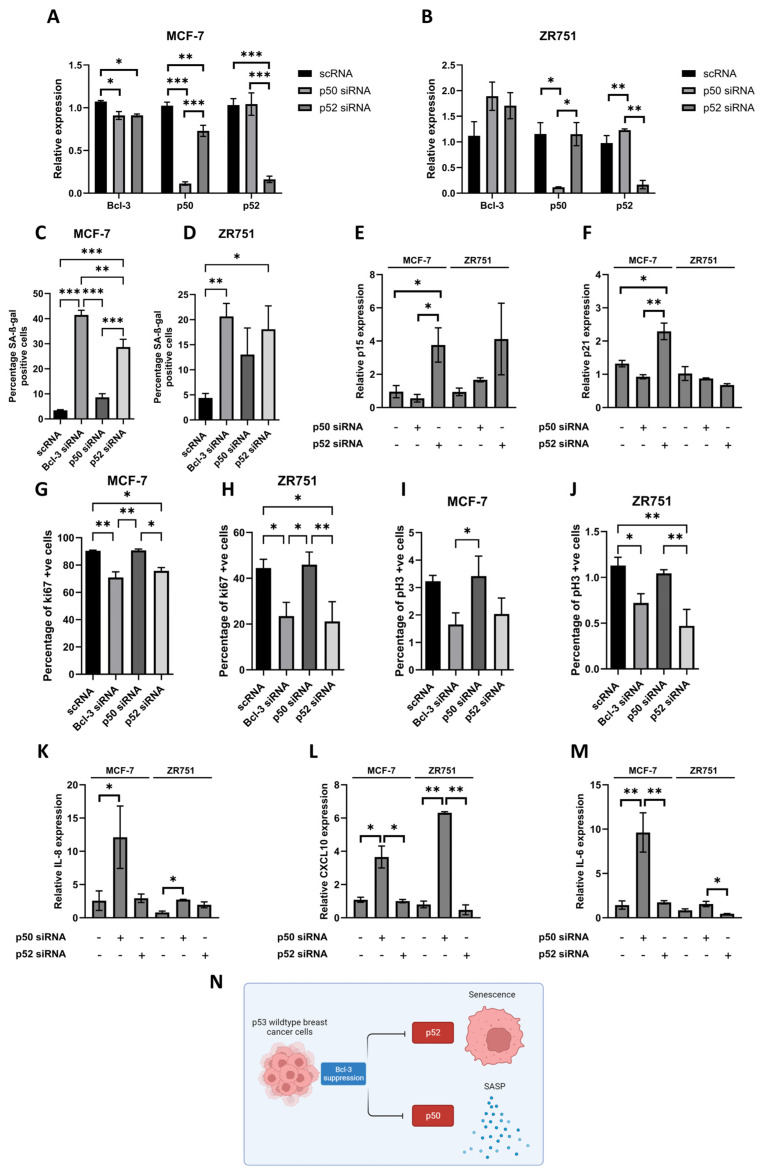
Loss of p52 and p50 induces senescence and SASP, respectively. NF-κB family members p50 and p52 were suppressed by siRNA for 6 days in (**A**) MCF-7 and (**B**) ZR751 cells and confirmed via qRT-PCR. (**C**,**D**) Senescence-associated β-gal staining was significantly increased following p52 suppression compared to that of scRNA controls in both cell lines in a similar response to that seen following Bcl3 knockdown. Analysis via qRT-PCR identified a significant increase in (**E**) p15 and (**F**) p21 expression in MCF-7 cells following p52 inhibition, with a similar trend in p15 expression also observed in ZR751 cells. The percentage of ki67- (**G**,**H**) and pH3- (**I**,**J**) positive cells was reduced in both cell lines treated with p52 siRNA in a similar manner to that of Bcl3 suppression, with no change observed following the suppression of p50. The expression of SASP genes (**K**) IL-6, (**L**) IL-8 and (**M**) CXCL10 was increased following p50 suppression and not following p52 knockdown. (**N**) Graphical illustration of how Bcl3 suppression in p53 wildtype breast cancer cells results in the induction of senescence and SASP through inhibiting p52 and p50 signalling, respectively. Error bars represent ± SEM of *n* = 3 with statistical differences determined using a one-way ANOVA; * *p* < 0.05, ** *p* < 0.01, and *** *p* < 0.01.

Both MCF-7 and ZR751 cells were treated with p50- or p52-specific siRNA for 6 days with knockdown efficiency confirmed via qRT-PCR (Figure 4A,B). Changes in Bcl3 expression were also tested with Bcl3 significantly reduced in MCF-7 cells following both p50 and p52 siRNA treatment (Figure 4A); however, this effect was not observed in ZR751 cells (Figure 4B). The suppression of p52 alone resulted in a significant increase in the percentage of SA-β-gal positive cells in both cell lines, in a similar response to what was observed with Bcl3 suppression (Figure 4C,D). The suppression of p50 also resulted in a small increase in SA-β-gal expression; however, this was not significant compared to scRNA-treated control cells. Quantitative PCR analysis demonstrated a significant increase in p15 and p21 expression in MCF-7 cells following the suppression of p52 and not p50, similarly to what was previously observed following Bcl3 suppression, although only p15 was upregulated in ZR751 cells (Figure 4E,F). A similar trend was also observed in the number of ki67- and pH3-positive cells with p52 suppression significantly reducing the number of proliferating cells in both cell lines in a similar manner to that of Bcl3 loss (Figure 4G–J). Together, these data suggest that the senescence phenotype following Bcl3 suppression was primarily due to disrupted p52-mediated transcription and not p50. In contrast, the suppression of p52 resulted in no change in SASP-related genes IL-6, IL-8 or CXCL10; however, all three were upregulated following the suppression of p50 (Figure 4K–M). Together, these data suggest that the disruption of p52 signalling is the main driver of the senescence phenotype observed following the suppression of Bcl3; however, the loss of Bcl3/p50 signalling may play an important role in maintaining this phenotype through the upregulation of SASP (Figure 4N).

### 3.5. Bcl3 Expression Is Associated with Relapse-Free Survival in p53 Mutant Breast Cancer

Having established the importance of Bcl3/NF-κB function in maintaining cell viability in breast cancer cell lines, we next sought to determine the clinical relevance of Bcl3 expression in breast cancer. Bcl3 is known to be overexpressed in human breast cancers and is associated with reduced metastasis-free survival [19,22]. Additionally, Bcl3 has previously been identified as a mediator of tumour cell survival following DNA damage in both p53-dependent and p53-independent contexts [29,30]. As normal p53 function is frequently lost in breast cancer, we began by determining whether or not the overexpression of Bcl3 negatively impacted the survival of patients receiving DNA-damaging agents and whether or not this depended on the p53 status of their tumours.

**Figure 5:**
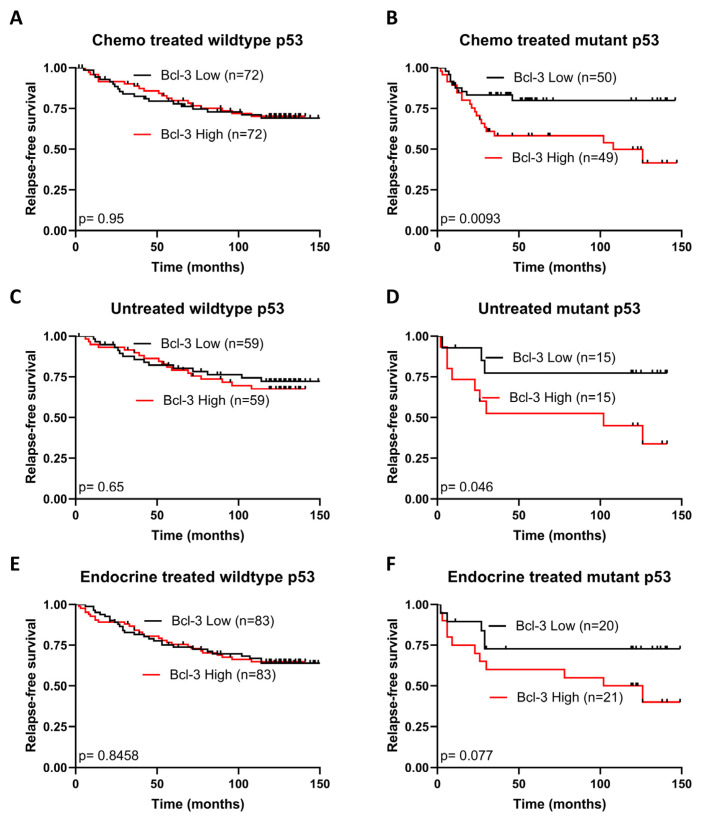
High Bcl3 expression is associated with reduced relapse-free survival in breast cancer patients with p53 mutations. The effect of high or low Bcl3 expression on RFS was assessed in clinical cohorts of breast cancer patients split based on the presence or absence of p53 mutations. (**A**) Graph showing that patients who received chemotherapy and maintained wildtype p53 had no difference in RFS; however, those with (**B**) p53 mutations and high levels of Bcl3 had significantly reduced RFS. A similar trend was observed in (**C**) p53 wildtype and (**D**) p53 mutant breast cancer patients who had not previously received any form of systemic treatment and in those that had received endocrine treatment (**E**,**F**). Analysis was performed on KM-plotter, which automatically calculates statistical significance using a log-rank test.

Given the importance of p53 in determining the response to chemotherapy, the effect of Bcl3 expression on RFS was assessed in patients that had received any form of systemic chemotherapy, with patients with wildtype or mutated p53 analysed separately. Patients with p53^mut^ tumours expressing high levels of Bcl3 showed significantly reduced RFS (*n* = 99, *p* = 0.0093) (Figure 5A), while no difference was observed in those with p53^wt^ tumours (*n* = 144, *p* = 0.95) (Figure 5B). This p53^mut^-dependent effect on Bcl3-associated survival was also observed in breast cancer patients who had not previously received any form of systemic treatment. Thus, a significant reduction in RFS was observed when Bcl3 was highly expressed in p53^mut^ tumours (*n* = 30, *p* = 0.046) (Figure 5C) while no difference was observed between p53^wt^ tumours expressing high and low levels of Bcl3 (*n* = 118, *p* = 0.65) (Figure 5D). A similar yet non-significant trend was also observed in endocrine-treated patients (p53^mut^ *n* = 41, *p* = 0.077 and p53^wt^ *n* = 166, *p* = 0.85) (Figure 5E,F). When each patient cohort was assessed without stratifying based on p53 status, no significant changes were observed (Supplementary Figure S5), further highlighting the close relationship between Bcl3 and mutated p53. 

These data suggest that overexpression of Bcl3 contributes to tumour progression in p53^mut^ breast cancers irrespective of prior chemotherapeutic intervention, suggesting that these patients may benefit most from Bcl3-targetting therapies, which based on our data presented here would have the additional benefit of inducing apoptosis and not senescence. 

## 4. Discussion

Despite the known role of Bcl3 in promoting breast cancer progression and metastasis [22,23], its role in regulating breast tumour cell survival without additional exogenous stress has remained unclear. In this study, we identify Bcl3/NF-κB dependence in ER-positive breast cancer cell lines and demonstrate the importance of Bcl3 in maintaining tumour cell survival. Bcl3 was suppressed using siRNA, which led to a significant increase in apoptosis in all breast cancer cell lines tested, whilst also inducing senescence in cell lines with functional p53. The importance of p53 in driving senescence was confirmed by simultaneously suppressing Bcl3 and p53, which rescued senescence but not the accompanying SASP phenotype, suggesting that Bcl3 loss mediated SASP gene expression independently of p53. Additional analysis demonstrated that senescence was largely a result of disrupted p52 signalling; however, the upregulation of SASP genes was mediated by a loss of p50. Comparisons of different clinical datasets demonstrated that high levels of Bcl3 only impacted RFS in patients also carrying p53 mutations, highlighting the importance of patient stratification for any future clinical use of Bcl3/NF-κB inhibitors. 

It has been previously established in breast and other cancer types that Bcl3 loss can sensitise tumour cells to exogenous stress such as DNA-damaging agents [29,45]. Additionally, Bcl3 loss can induce apoptosis in normal mammary gland development [21], while Bcl3/ NF-κB activity can promote tumour survival in colorectal cancer cells through AKT activation [46]. Here, we show that Bcl3 loss induces apoptosis in breast cancer cell lines without the need for additional exogenous stress, while also inducing senescence in cancer cells that maintain functional p53. The role of Bcl3 in regulating senescence in cancer is not well understood, with only one study on liver cancer demonstrating a role for Bcl3 in regulating SASP factors [47]. Our findings show for the first time that loss of Bcl3 in tumour cells can induce senescence and upregulate the accompanying SASP cytokines that are a hallmark of senescence. Similar studies in primary human fibroblasts have shown that the suppression of either Bcl3 or p52 can induce senescence through stabilising p53 and p21 proteins as well as regulating EZH2 expression [48], which supports our data suggesting that senescence was driven specifically through p52 loss. Interestingly, the production of reactive oxygen species (ROS) mediated by ras-related C3 botulinum toxin substrate 1 (RAC1) and Cell Division Cycle 42 (CDC42) also contributed to senescence in fibroblasts [48]. Although this was not investigated in this study, our previous work has shown that a loss of Bcl3 can regulate CDC42 transcription in breast cancer cell lines, including MCF-7 cells [24], which could also contribute to ROS production and senescence. Bcl3 loss has also been shown to stabilise p53 in MCF-7 cells following DNA damage through the downregulation of MDM2, however in this context resulting in the induction of apoptosis and not senescence [29]. Although, Bcl3 can regulate cell death through p53-independent pathways [30,33], it appears that p53 and non-canonical NF-κB-mediated transcription are crucial for its role in regulating senescence. Our data, particularly the observation that p53 loss suppressed Bcl3 expression in MCF-7 cells, further highlight the complex relationship between Bcl3/NF-κB and p53 that has been previously suggested [49]. Further work is now required to fully characterise this pathway and to investigate how this could be exploited therapeutically. 

Although senescence induction was a result of deregulated non-canonical NF-κB signalling, the accompanying SASP phenotype was driven through a loss of canonical p50 activity. The SASP phenotype is largely attributed to maintaining senescence; however, it has been implicated as tumour-promoting in certain contexts [50]. Key canonical NF-κB member, RelA/p65, is a master regulator of SASP [43] and commonly dimerises with p50 [51], while p50 homodimers have been shown to bind to the same sites as p65 homodimers and p50/p65 heterodimers [52] and can directly regulate IL-6, IL-8 and CXCL10 expression [53,54]. Our data further support the importance of p50 in regulating the transcription of SASP factors and demonstrate the distinct regulation of these independent of p53, unlike the induction of senescence. This could have significant clinical implications as the use of TIS as a clinical entity remains controversial, largely due to the tumour-promoting properties of SASP cytokines such as IL-6 in certain contexts [50,55]. Targeting non-canonical NF-κB could be used to overcome this issue by inducing senescence without stimulating SASP.

The induction of apoptosis through targeting Bcl3 represents a more conventional therapeutic approach that could be particularly beneficial for patients with high Bcl3 expression and a p53 mutation. Around 30% of all breast cancer cases carry a p53 mutation, with the highest rates observed in hard-to-treat triple-negative breast cancers (TNBC) [56,57]. Recently, a small-molecule Bcl3 inhibitor was shown to significantly inhibit TNBC tumour growth in vivo through the induction of apoptosis [32]. Despite this, Bcl3 loss in two transgenic models of breast cancer resulted in no change to primary tumour growth, but affected progression to metastasis [22,23], suggesting that Bcl3 may be more important in established tumours than tumourigenesis in the p53^wt^ setting. Colorectal cancer cells can develop an ‘addiction’ to Bcl3 and are therefore primed to undergo apoptosis in response to Bcl3 suppression [33]. A similar phenomenon could be true for breast cancer; however, further characterisation is required, with the effects of Bcl3 inhibition on patient-derived tumour models of different p53 statuses being particularly interesting. Bcl3 inhibition should also be investigated in the context of tamoxifen resistance, which has been associated with high levels of Bcl3 expression [38].

## 5. Conclusions

In conclusion, our findings establish Bcl3 as an important regulator of breast cancer cell survival, highlighting its regulation of the NF-κB signalling network as an important factor in maintaining survival. We show that the suppression of Bcl3 causes a loss of viability primarily through the induction of apoptosis and the additional induction of senescence in breast cancer cell lines that maintain wildtype p53. These data could help direct future clinical approaches with patients overexpressing Bcl3 and harbouring p53 mutations being most likely to benefit from Bcl3 inhibition. 

## Data Availability

Data are contained within the article or supplementary material. Raw data files for figures presented in this study are available on request.

## Supplementary Materials

The following supporting information can be downloaded athttps://www.mdpi.com/article/10.3390/biomedicines12010143/s1. Figure S1: Bcl-3 suppression increases apoptosis in breast cancer cell lines; Figure S2: Bcl-3 suppression reduces the percentage of ki67 and pH3 positive MCF-7 cells; Figure S3: Bcl-3 suppression increases SA-β-gal staining in p53 wild type breast cancer cell lines; Figure S4: Additional loss of p53 following Bcl-3 suppression does not increase apoptosis; Figure S5: Bcl-3 expression does not impact RFS in breast cancer patients without further stratification.
