# Supplementary material for: Suppression of Bcl3 Disrupts Viability of Breast Cancer Cells through Both p53-Dependent and p53-Independent Mechanisms via Loss of NF-κB Signalling
Source: Biomedicines. 2024 Jan 10;12(1):143. doi: 10.3390/biomedicines12010143 (PMC10813424; doi:10.3390/biomedicines12010143)
Supplement: Supplementary file 1 [file biomedicines-12-00143-s001.zip › Slide1.pdf]

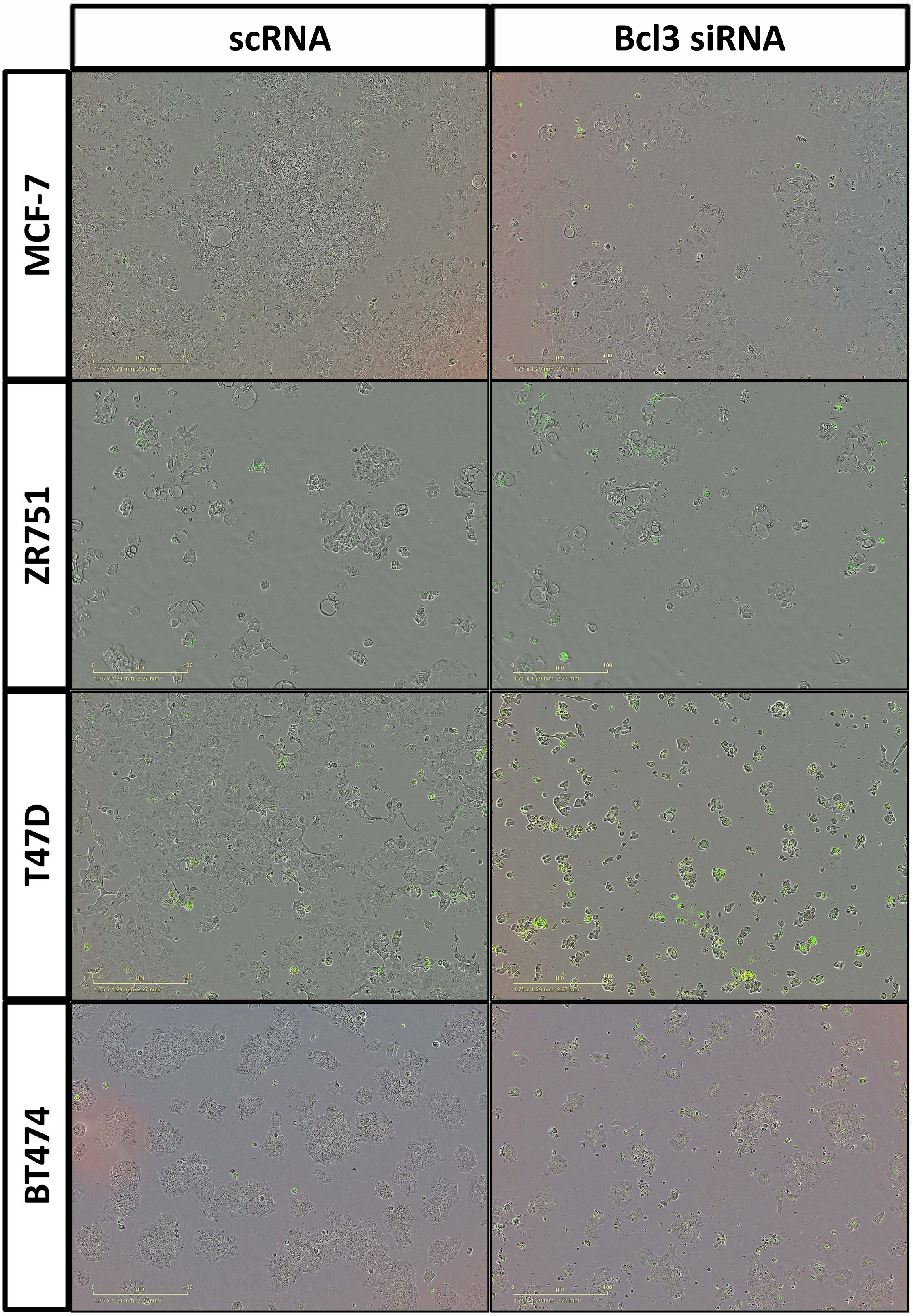

Supplementary Figure S1- Bcl-3 suppression increases apoptosis in breast cancer cell lines. Representative images of IncuCyte annexin V staining in MCF-7, ZR751, T47D and BT474 cells following 6 days of Bcl-3 siRNA treatment.
