# Supplementary material for: Suppression of Bcl3 Disrupts Viability of Breast Cancer Cells through Both p53-Dependent and p53-Independent Mechanisms via Loss of NF-κB Signalling
Source: Biomedicines. 2024 Jan 10;12(1):143. doi: 10.3390/biomedicines12010143 (PMC10813424; doi:10.3390/biomedicines12010143)
Supplement: Supplementary file 1 [file biomedicines-12-00143-s001.zip › Slide2.pdf]

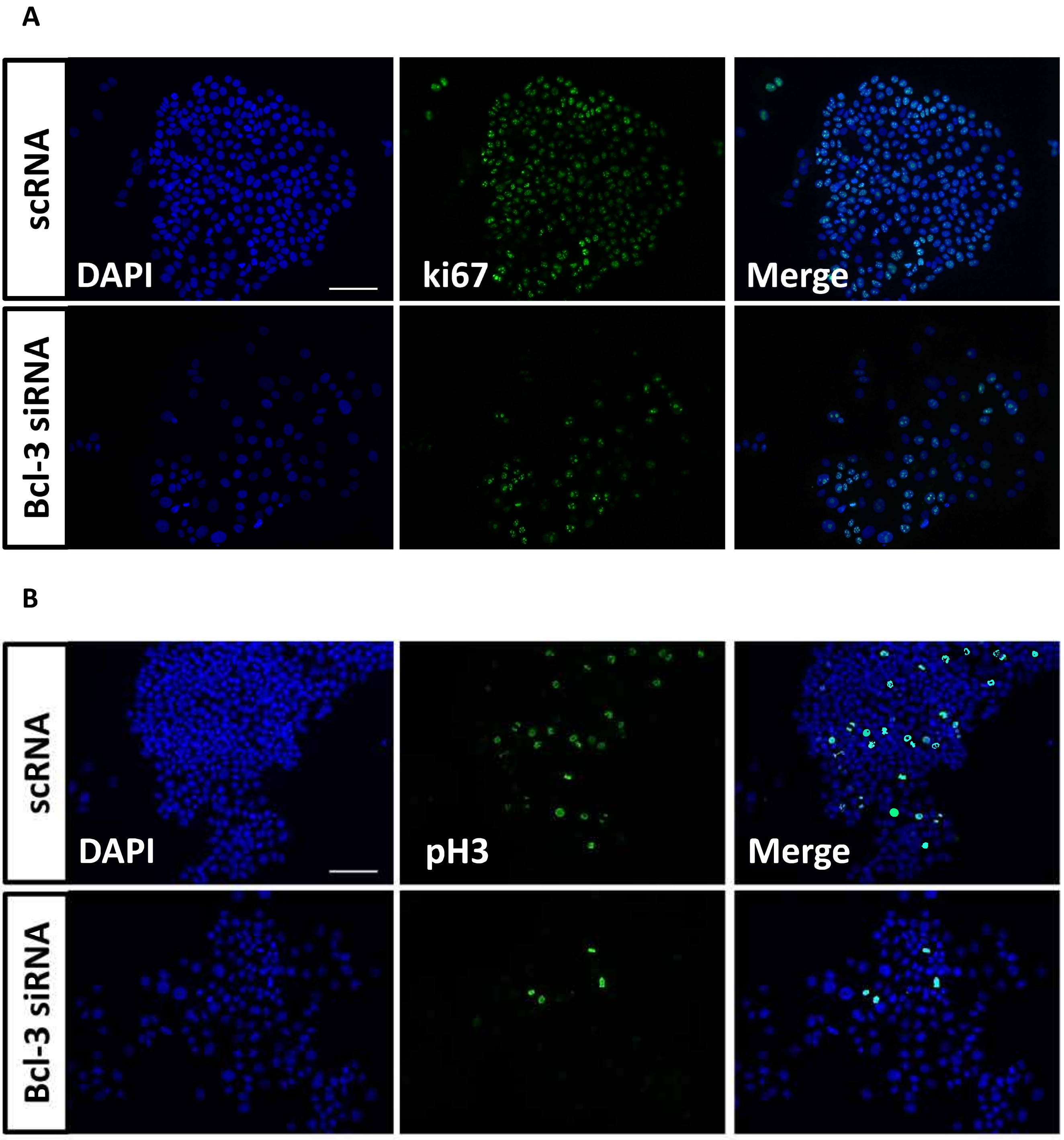


**Supplementary Figure S2- Bcl-3 suppression reduces the percentage of ki67 and pH3 positive MCF-7 cells.** Representative images of MCF-7 cells stained for either **(A)** ki67 or **(B)** pH3 following 6 days of Bcl-3 siRNA treatment. Scale bar = 75 μm.
