# Supplementary material for: Suppression of Bcl3 Disrupts Viability of Breast Cancer Cells through Both p53-Dependent and p53-Independent Mechanisms via Loss of NF-κB Signalling
Source: Biomedicines. 2024 Jan 10;12(1):143. doi: 10.3390/biomedicines12010143 (PMC10813424; doi:10.3390/biomedicines12010143)
Supplement: Supplementary file 1 [file biomedicines-12-00143-s001.zip › Slide4.pdf]

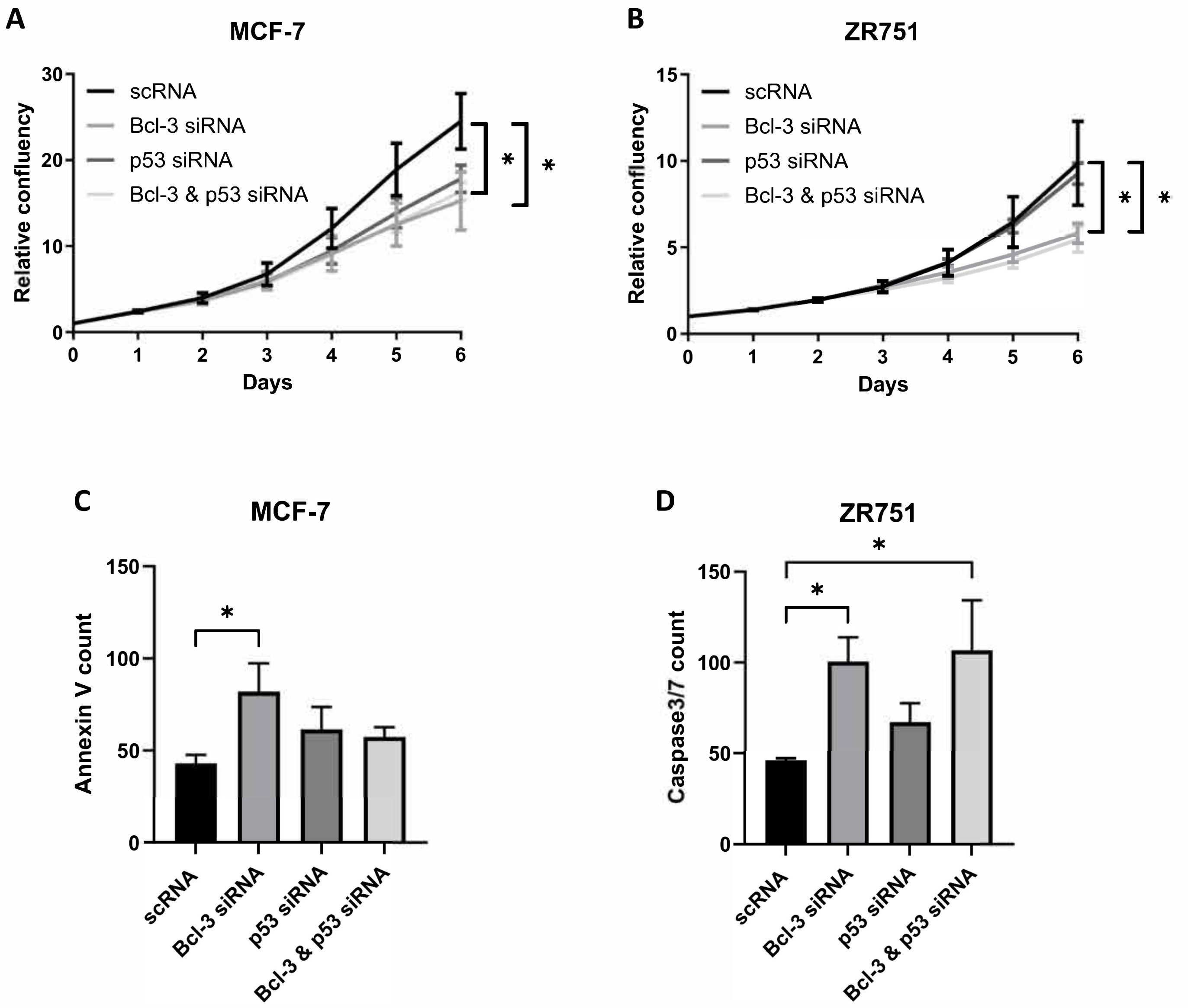

Supplementary Figure S4- Additional loss of p53 following Bcl-3 suppression does not increase apoptosis. IncuCyte analysis of **(A)** MCF-7 and **(B)** ZR751 cells following siRNA suppression of Bcl-3 and/or p53 showed no additional loss in confluency when Bcl-3 and p53 were simultaneously suppressed compared to Bcl-3 alone. **(C)** Annexin V counts in MCF-7 cells and **(D) c**aspase 3/7 counts in ZR751 cells 6 days post siRNA transfection were not increased with additional p53 suppression.
