# Supplementary material for: Suppression of Bcl3 Disrupts Viability of Breast Cancer Cells through Both p53-Dependent and p53-Independent Mechanisms via Loss of NF-κB Signalling
Source: Biomedicines. 2024 Jan 10;12(1):143. doi: 10.3390/biomedicines12010143 (PMC10813424; doi:10.3390/biomedicines12010143)
Supplement: Supplementary file 1 [file biomedicines-12-00143-s001.zip › Slide5.pdf]

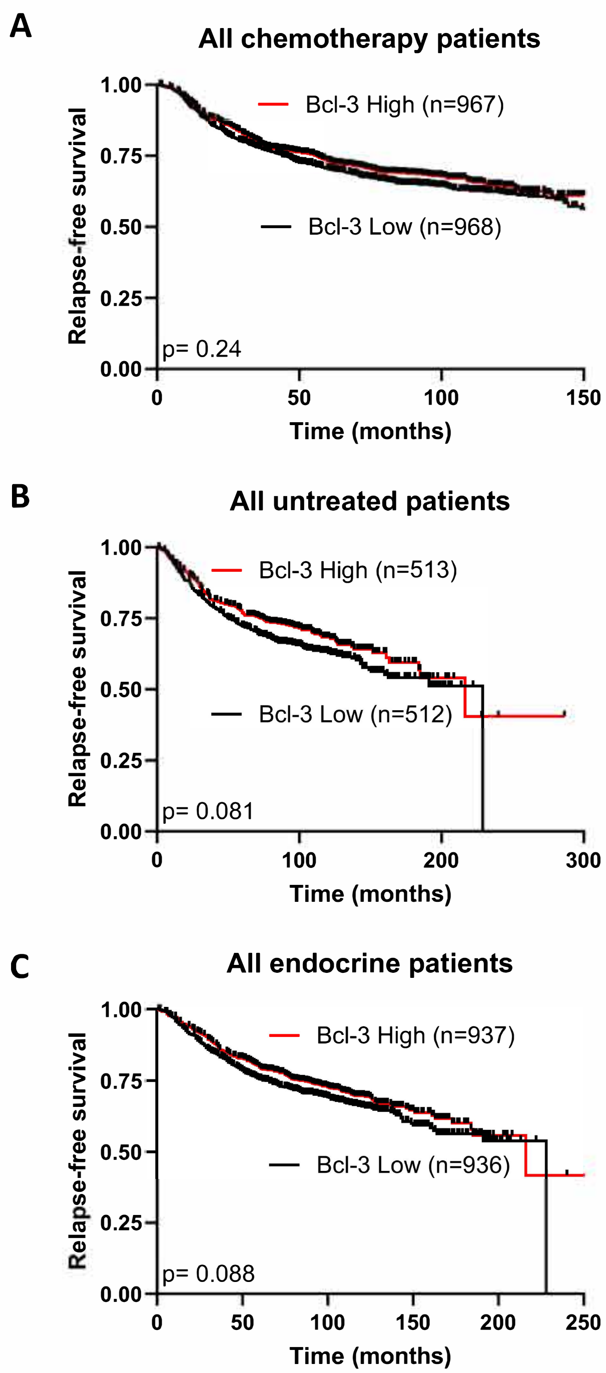

Supplementary Figure S5- Bcl-3 expression does not impact RFS in breast cancer patients without further stratification. The effect of high or low Bcl-3 expression on RFS was assessed in clinical cohorts of breast cancer patients that had either received **(A)** chemotherapy **(B)** no systemic treatment or **(C)** endocrine treatment. Analysis was performed on KM-plotter which automatically calculates statistical significance using log-rank test.
